# Increased risk of vertebral fractures and reduced risk of femur fractures in patients with chronic hypoparathyroidism: a nationwide cohort study in Sweden

**DOI:** 10.1093/jbmr/zjaf061

**Published:** 2025-05-05

**Authors:** Sigridur Björnsdottir, Wafa Kamal, Michael Mannstadt, Outi Mäkitie, Tim Spelman, Olle Kämpe, Bente L Langdahl

**Affiliations:** Department of Molecular Medicine and Surgery, Karolinska Institutet, 171 76 Stockholm, Sweden; Department of Molecular Medicine and Surgery, Karolinska Institutet, 171 76 Stockholm, Sweden; Department of Endocrinology, Metabolism and Diabetes, Karolinska University Hospital, 171 76 Stockholm, Sweden; Endocrine Unit, Massachusetts General Hospital and Harvard Medical School, Boston, MA 02114, United States; Children's Hospital, Pediatric Research Center, University of Helsinki and Helsinki University Hospital, FI-00290, Helsinki, Finland; Clinical Genetics, Karolinska University Hospital, Stockholm, Sweden; Department of Clinical Neuroscience, Karolinska Institutet, 171 76, Stockholm, Sweden; Department of Endocrinology, Metabolism and Diabetes, Karolinska University Hospital, 171 76 Stockholm, Sweden; Department of Medicine, Solna, Karolinska Institutet, 171 76, Stockholm, Sweden; Endocrinology and Internal Medicine, Aarhus University Hospital and Clinical Medicine, 8200, Aarhus University, Aarhus, Denmark

**Keywords:** osteoporosis, fractures, hypoparathyroidism, epidemiology, Sweden

## Abstract

Patients with chronic hypoparathyroidism (hypoPT) have reduced bone remodeling, leading to increased bone density and abnormalities in microarchitecture and bone strength. Whether these patients have an increased fracture risk remains unclear. This study aimed to evaluate the risk of major osteoporotic fracture (MOF), osteoporosis diagnoses, and osteoporosis medication use in patients with chronic hypoPT in Sweden. Subtypes of fractures were also assessed. Using the Swedish National Patient Register, the Prescribed Drug Register, and the Total Population Register, we identified 1915 patients with chronic hypoPT treated with active vitamin D between 1997 and 2018, and 15 838 matched controls. After adjustment, patients with chronic hypoPT did not have a higher risk of MOF compared to controls (HR 0.93; 95% CI: 0.69-1.26). However, they had a higher risk of vertebral fractures (HR 1.55; 95% CI: 1.12-2.14) and a lower risk of femur fractures (HR 0.70; 95% CI: 0.50-0.98) compared to controls. They were more often diagnosed with osteoporosis (HR 1.54; 95% CI: 1.21-1.95) but less frequently prescribed osteoporosis medication (HR 0.69; 95% CI: 0.54-0.88) compared to controls. No difference in the MOF risk was observed between females and males (*p* for interaction = 0.872) or between patients with surgical and non-surgical chronic hypoPT (*p* for interaction = 0.072). In this large Swedish cohort, chronic hypoPT was not associated with an increased risk of MOF. Vertebral fracture risk was higher, while the femur fracture risk was lower compared to controls. Despite higher prevalence of osteoporosis diagnoses, these patients received less frequently osteoporosis medications.

## Introduction

Hypoparathyroidism (hypoPT) is a rare disease, with anterior neck surgery being the leading cause, accounting for approximately 75% of cases.^1^ Other causes include genetic disorders, autoimmune destruction of the parathyroid glands, infiltrative disorders, and exposure to ionizing radiation. HypoPT is characterized by low serum calcium levels and an inappropriately low production of PTH.^1^ Conventional treatment of hypoPT consists of active vitamin D analogs and oral calcium supplementation. The most common definition of chronic postsurgical hypoPT is the presence of the disease 12 mo after surgery.^2–5^

PTH plays a crucial role in regulating bone structure and remodeling.^6^ By stimulating RANK-ligand production by the osteoblastic cells, PTH activates osteoclasts and thereby promotes bone remodeling. Chronic PTH deficiency leads to a significant reduction in bone turnover. Patients with hypoPT treated with conventional therapy have increased bone mass.^7^ Histomorphometry of b1 biopsies from these patients has shown increased b1 volume and reduced remodeling activity and alterations in microarchitecture.^7^^,^^8^ A study using HRpQCT of the radius and tibia has shown increased cortical and trabecular bone mass.^9^ Interestingly, one recent study showed that the bone material strength index, as measured by in vivo microindentation at the anterior tibia, was lower in patients with hypoPT compared with controls.^10^ While the increased bone mass in patients with chronic hypoPT might suggest greater fracture resistance, the markedly reduced bone turnover may lead to more mature but brittle bone. However, it remains unclear whether these alterations have an overall negative or positive effect on bone strength. A key measure of bone strength in patients with hypoPT is the assessment of fracture risk.

Existing studies are conflicting, as some have shown increased and others decreased fracture risk in patients with hypoPT.^11–13^ The rarity of the disease has limited prior studies to small patient cohorts, which may partly explain these inconsistencies. Therefore, investigating fracture risk in larger patient populations is essential.

By merging data from population-based registers in Sweden, we were able to identify a large cohort of patients with chronic hypoPT receiving conventional treatment. This enabled us to investigate the risk of major osteoporotic fracture (MOF), the prevalence of osteoporosis diagnoses, and the dispensation of osteoporosis medication compared to matched controls. Additional endpoints included fractures by specific anatomical locations.

## Materials and methods

### Registries

The Swedish National Patient Register (SNPR) includes hospital inpatient data since 1964, with nationwide coverage established in 1987, and hospital outpatient data since 2001. While it does not capture data from primary care settings, most patients with chronic hypoPT in Sweden are managed through hospital outpatient clinics. The SNPR includes information on age, sex, dates of hospital admission and discharge, surgical procedure codes, and discharge diagnoses. Reporting to the SNPR is mandatory for all healthcare providers.^14^ Data from the SNPR can be linked to other registries through the unique personal identity number assigned to all Swedish residents.

The Swedish Prescribed Drug Register (SPDR) has collected data on all prescription drugs dispensed to the Swedish population since July 2005.^15^ It includes data on prescriptions from primary care and classifies medications using the Anatomical Therapeutic Chemical (ATC) classification system. Registration of all prescribed and dispensed drugs is mandatory for pharmacies in Sweden. However, the SPDR does not cover over-the-counter medications, drugs used in hospital settings, or clinical information on diagnoses or treatment indications. The Swedish Total Population Register collects demographic data on the population.^16^

### Participants

This registry-based study was approved by the Research Ethics Board of Stockholm, Sweden (approval no. DNR: 2017/476-31/4). We used the SNPR to identify individuals with the International Classification of Diseases (ICD)-10 diagnosis of hypoPT (ICD-10: E20.0, E20.2-9), postsurgical hypoPT (ICD-10: E89.2), DiGeorge syndrome (ICD-10 D82.1), and autoimmune polyglandular failure (ICD-10: E31.0) between January 1997 and December 2018. This interval was chosen as specific ICD-10 codes for hypoPT were introduced in 1997. The SPDR that started in 2005 was used to enhance the diagnostic accuracy by including only patients with at least 2 dispensations of active vitamin D (dihydrotachysterol (ATC A11CC02), alfacalcidol (ATC A11CC03), or calcitriol (ATC A11CC04)) with or without calcium supplements. For those diagnosed from 2005 and onward, these dispensations were required at least 13 mo post-diagnosis. For those diagnosed before 2005, at least 2 dispensations of active vitamin D were required within the first year of SPDR. This approach ensured the inclusion of patients with chronic, not transient, hypoPT.

Patients were excluded if they had fewer than 2 dispensations of active vitamin D during the final year of follow-up. To further improve the diagnosis precision, we excluded patients and their controls with kidney failure at baseline or within 1 yr from baseline (ICD codes provided in Table S1), as these individuals are often treated with active vitamin D for secondary hyperparathyroidism and may have been incorrectly coded for hypoPT.

**Figure 1:**
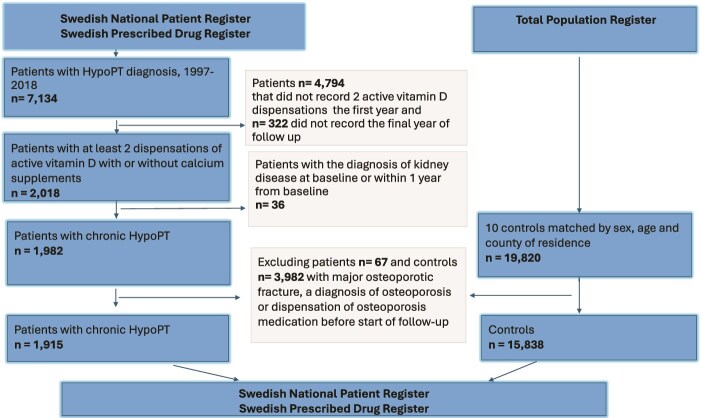
Flowchart of study participants.

For each patient, we randomly identified 10 controls matched by year of birth, sex, and county of residence using the Register of Population, including all Swedish residents alive at the end of each year (Figure 1). Patients and controls with a history of MOF, a prior diagnosis of osteoporosis, or dispensation of osteoporosis medication before start of follow-up were subsequently excluded (Figure 1). The follow up for cases and matched controls began 13 mo after the first recorded hypoPT diagnosis in the SNPR for individuals diagnosed in 2005 or later. For those with an initial hypoPT diagnosis in the SNPR prior to 2005, follow-up started at the time of the second dispensation of active vitamin D in the drug register. Follow-up ended on December 31, 2018, or the date of last recorded activity in the registries, whichever occurred first.

The diagnostic criteria used in our study have been validated previously.^16^ In this validation study, Swedish hypoPT patients were included based on register data and the diagnosis was then verified through medical chart review in a subset of patients. Patients were included if they had a diagnosis of hypoPT reported to the National Patient Register and had received 3 or more dispensations of conventional treatment during the first year after diagnosis or had 2 dispensations per year in 2 consecutive years. The positive predictive value of correct diagnosis was 91% in 120 randomly selected cases when the diagnosis was verified by chart review.

### Comorbidities

We identified the ICD-10 codes for thyrotoxicosis, type 1 and type 2 diabetes, rheumatoid arthritis, liver disease, high alcohol consumption, chronic kidney disease, malignant neoplasms, cerebrovascular disease, and ischemic heart disease prior to the start of follow-up. As a proxy for heavy smoking, we used the ICD-10 code for chronic obstructive pulmonary disease (COPD) and/or the ATC code for drugs used in nicotine dependence. For high alcohol consumption, we used the ICD-10 codes for alcohol related disorders and/or the ATC-code for drugs prescribed for alcohol dependence (ICD-10 and ATC-codes provided in Table S2). These comorbidities, which are known causes of secondary osteoporosis and are included in the Fracture Risk Assessment Tool (FRAX),^17^ were adjusted for in the analysis.

### Outcome measures

The primary outcome was the risk of MOF, which includes hip, vertebrae, humerus, or forearm fractures identified through ICD-10 codes in the patient register (ICD-10 codes listed in Table S3). Secondary outcomes included the diagnosis of osteoporosis (ICD-10 codes: M80 for osteoporosis with pathological fracture and M81 for osteoporosis without pathological fracture) and the dispensation of osteoporosis medication (ATC codes provided in Table S4).

Subgroup analyses were conducted on patients and controls with an incident fracture. Additionally, we evaluated the risk of MOF separately in surgical vs non-surgical chronic hypoPT, females vs males with chronic hypoPT, and in age groups <50 and ≥50 yr old.

### Statistical analysis

Categorical variables were summarized using frequency and percentage. Continuous variables were summarized using mean and SD or median and interquartile range (IQR) as appropriate. A McNemar chi-square test was used to compare risk factors at baseline between cases and their matched controls. Multivariable marginal Cox regression models were used to estimate the adjusted hazard ratios (HRs) with 95% CIs for the associations between chronic hypoPT and the incident primary MOF, a diagnosis of osteoporosis, and dispensation of osteoporosis medication endpoints. Additional endpoints included vertebral, pelvic, femur, proximal humerus, and forearm fractures. Subgroup analyses were done based on sex, age, and surgical status. For all outcomes, adjustments were made for the following risk factors for MOF: thyrotoxicosis, rheumatoid arthritis, history of diabetes, COPD (as a proxy for heavy smoking), liver disease, alcohol related disorders, CKD, malignant neoplasms, cerebrovascular diseases, ischemic heart disease, age, sex, and prior thyroxine use as indicated.

Hazard proportionality was assessed via analysis of scaled Schoenfeld residuals. An interaction term between sex, surgical and non-surgical causes, and hypoPT was separately included in the multivariable Cox regression models to identify whether the association between hypoPT and outcomes varied across different disease etiology subgroups. All multivariable models were assessed for multicollinearity and heteroskedasticity.

A post-hoc power analysis estimated that the final sample of 1915 cases and 15 838 controls had 95% power to detect a minimum difference of 2% in the proportion of cases and controls with a fracture, presuming a baseline population prevalence of 5%. If a prevalence of 1% is presumed, the sample had 95% power to detect a minimum difference of 1.2%. Ultimately, our study observed an absolute effect size of 0.9% as statistically significant in the case of both vertebral fractures and femur fractures.

For all analyses, a *p* < .05 was considered statistically significant. All analyses were conducted using Stata version 16.1 (StataCorp) and R version 4.0.5 (R Foundation for Statistical Computing).

## Results

### Patient characteristics

Using our strict criteria, we identified 1982 patients with chronic hypoPT and 19 820 controls matched by sex, age, and county of residence. After excluding cases (*n* = 67) and controls (*n* = 3982) with prior MOF, osteoporosis diagnoses or dispensations of osteoporosis medication, a total of 1915 cases and 15 838 controls were alive at the start of follow-up and included in the study (Figure 1). Among the chronic hypoPT cases, 72% were due to surgery, the mean age was 53.9 (SD 19.7), and 1457 (76.1%) were females. The median follow-up time was 9.3 (IQR 5.0-15.2) yr for cases and 10.4 (IQR 5.2-17) yr for controls (Table 1). Compared to controls, the chronic hypoPT cohort had significantly more women, were older, had a shorter follow-up time, and generally exhibited more comorbidities (Table 1). Additionally, a significantly higher proportion of women with chronic hypoPT had pre-baseline prescription of hormone replacement therapy compared to control women (10.0% vs 5.6%), *p* < .001.

**Table 1:** Baseline characteristics of patients with chronic hypoparathyroidism and matched controls after excluding patients and controls with major osteoporotic fractures, a diagnosis of osteoporosis, or dispensation of osteoporosis medication before the start of follow-up.

	**Chronic hypoparathyroidism**	**Controls**	***p*-value**
**No. of patients**	1915	15 838	
**Age (yr) mean (SD)**	53.9 (19.7)	50.2 (18.6)	**<.001**
**Women, *n* (%)**	1457 (76.1)	11 606 (73.3)	**.009**
**Follow-up time, yr (Median, IQR)**	9.3 (5.0–15.2)	10.4 (5.2–17.0)	**<.001**
**Outpatient visits per yr (Median, IQR)**	3.6 (2.1–6.5)	N/A	
**Etiology**			
** Postsurgical hypoparathyroidism**	1423 (71.8)	N/A	
**DiGeorge syndrome**	27 (1.4)	N/A	
**Idiopathic hypoparathyroidism**	245 (12.4)	N/A	
**Other or unspecified hypoparathyroidism**	265 (13.4)	N/A	
**Dispensations of active vitamin D per year (Median, IQR)**	6.1 (4.3–10.1)	N/A	
**Dispensations of calcium supplementation per year (Median, IQR)**	3.7 (0.2–9.3)	N/A	
**Comorbidities, *n* (%)**			
**Thyrotoxicosis**	310 (16.2)	35 (0.2)	**<.001**
**Rheumatoid arthritis**	7 (0.4)	30 (0.2)	0.11
**Diabetes**			
**Type 1 diabetes**	39 (2.0)	121 (0.8)	**<.001**
**Type 2 diabetes**	78 (4.1)	253 (1.6)	**<.001**
**Chronic obstructive pulmonary disease**	35 (1.8)	103 (0.7)	**<.001**
**Liver disease**	14 (0.7)	33 (0.2)	**<.001**
**Alcohol related disorders**	24 (1.3)	161 (1.0)	.335
**Chronic kidney disease**	0 (0.0)	27 (0.2)	.11
**Malignant neoplasms**	287 (15)	435 (2.8)	**<.001**
**Cerebrovascular disease**	76 (4.0)	240 (1.5)	**<.001**
**Ischemic heart disease**	117 (6.1)	452 (2.9)	**<.001**
**Epilepsy**	43 (2.3)	110 (0.7)	**<.001**

Bold values indicate statistical significance (*p* < 0.05).Values for continuous data are mean ± SD or median (IQR) and count (%) for categorical data.

Abbreviations: IQR, interquartile range; N/A, not applicable.

### Outcomes

**Figure 2:**
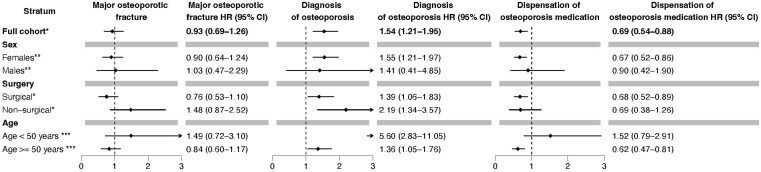
The risk of major osteoporosis fracture, diagnosis of osteoporosis, and dispensation of osteoporosis medication in patients with chronic hypoPT compared to controls, including subgroup analyses. ^*^Adjusted for comorbidities, age, and sex. ^**^Adjusted for comorbidities and age*.*  ^***^Adjusted for comorbidities and sex.

After adjustment for comorbidities, age, and sex, patients with chronic hypoPT did not have an increased risk of MOF (HR 0.93; 95% CI: 0.69-1.26) compared to controls (Figure 2). However, they had higher risk of vertebral fracture (HR 1.55; 95% CI: 1.12-2.14) and a lower risk of femur fractures (HR 0.70; 95% CI: 0.50-0.98) compared to controls (Table 2).

**Table 2:** The risk of fractures (HR (95% CI)) in patients with chronic hypoparathyroidism compared to controls.

**Fractures** ^**a**^ **, *n* (%)**	**Cases (*n* = 1915)**	**Controls (*n* = 15 838)**	**HR (95% CI)**	***p*-value**
				
Vertebral fractures	49 (2.6)	267 (1.7)	1.55 (1.12, 2.14)	**.008**
Pelvic fractures	11 (0.6)	94 (0.6)	0.92 (0.48, 1.78)	.809
Femur	39 (2.0)	460 (2.9)	0.70 (0.50, 0.98)	**.038**
Proximal humerus	25 (1.3)	309 (2.0)	0.69 (0.46, 1.06)	.09
Forearm	57 (3.0)	577 (3.6)	0.94 (0.71, 1.25)	.676

a Adjusted for comorbidities, age, and sex. Bold values indicate statistical significance (*p* < 0.05).

Patients with vertebral fractures were more likely to be women (*p* = .026), older (*p* < .001) and had a higher prevalence of epilepsy diagnoses (*p* = .008) compared to patients without vertebral fractures (Table 3). There was no significant difference in the dispensation of horm1 replacement therapy between patients with or without vertebral fractures. There was no significant difference in vertebral fracture risk between patients with surgical and non-surgical chronic hypoPT. Patients with chronic hypoPT were more frequently diagnosed with osteoporosis (HR 1.54; 95% CI: 1.21-1.95; *p* < .001) but were less likely to receive osteoporosis medications (HR 0.69; 95% CI: 0.54-0.88; *p* = .002) compared to controls.

**Table 3:** Factors influencing the risk of vertebral fractures in patients with chronic hypoparathyroidism.

**Factor/Category**	**Cases with vertebral fractures (*n* = 49)**	**Cases without vertebral fractures (*n* = 1866)**	***p*-value** ^**a**^
**Age, yr (mean (SD))**	53.86 (19.71)	50.20 (18.55)	**<.001**
**Sex *n* (%)**			
Females	44 (89.8)	1413 (75.7)	**.026**
Males	5 (10.2)	453 (24.3)	
**Comorbidities (any pre-baseline record) *n* (%)**			
Thyrotoxicosis	4 (8.2)	306 (16.4)	.167
T1DM	0 (0.0)	39 (2.1)	.623
T2DM	0 (0.0)	78 (4.2)	.263
Other diabetes	0 (0.0)	0 (0.0)	N/A
Rheumatoid arthritis with Rh factor	1 (2.0)	6 (0.3)	.166
Other rheumatoid arthritis	1 (2.0)	13 (0.7)	.305
Liver disease	1 (2.0)	13 (0.7)	.305
Alcohol related disorders	0 (0.0)	24 (1.3)	1.000
Chronic kidney disease	0 (0.0)	0 (0.0)	N/A
Malignant neoplasms	6 (12.2)	281 (15.1)	.586
COPD	3 (6.1)	32 (1.7)	.058
Cerebrovascular disease	2 (4.1)	74 (4.0)	1.000
Ischaemic heart disease	5 (10.2)	112 (6.0)	.220
**Any pre-baseline estrogen dispensation**	4 (8.2)	187 (10.0)	.813
**Any estrogen dispensation ever** ^**b**^	18 (36.7)	506 (27.1)	.145
**Any epilepsy episode ever** ^**b**^	6 (12.2)	80 (4.3)	**.008**
**Any use of anti-epileptics ever** ^**b**^	2 (4.1)	87 (4.7)	1.000

^a^ Rank-sum, chi-square or Fisher’s exact as appropriate.

^b^ Any time pre-baseline or during the patient-level post-baseline period. Bold values indicate statistical significance (*p* < 0.05).

### Subgroup analysis

In the subgroup of patients and controls with any incident fracture, a significantly smaller proportion of cases received at least 1 post-baseline dispensation of osteoporosis medication (30/295 = 10.2%) compared to controls (416/2509 = 16.6%), *p* = .004. Among those without an osteoporosis medication dispensation within 2 yr of MOF, chronic hypoPT cases were significantly more likely to be on hormone replacement therapy compared to controls (12.8% vs 3.9%; *p* = .008).

There was no significant difference in the risk of MOF between females and males with chronic hypoPT (*p* for interaction = 0.872) or between patients with surgical and non-surgical chronic hypoPT (*p* for interaction = 0.072).

### Sensitivity analyses

To test for a potential ascertainment bias due to differential follow-up in cases and their matched controls, we ran a sensitivity analysis of the primary MOF endpoint using simultaneous censoring of the matched case-control unit. This involved censoring all members of the matched unit at the point of earliest follow-up end of any member of the matched unit. Only a very small shift in HR was observed, from 0.93 (95% CI: 0.69, 1.26) in the original model to HR 0.89 (95% CI: 0.63, 1.27) in the simultaneously censored model. This suggests that any impact of differential follow-up on the primary MOF outcome was negligible. As a further sensitivity analysis, we extended the primary multivariable MOF model to adjust for baseline differences in comorbid epilepsy between cases and controls. This had a minimal effect on the model results, recording an almost identical HR of 0.94 (95% CI: 0.74, 1.20).

## Discussion

This large register-based study, which included 1915 patients with chronic hypoPT and 15 838 matched controls, found no increased risk of MOF in patients with chronic hypoPT in Sweden. However, these patients demonstrated a higher risk of vertebral fractures, but a lower risk of femur fractures compared to controls. Additionally, they were more frequently diagnosed with osteoporosis (ICD diagnosis) but were less likely to receive osteoporosis medications compared to controls. In subgroup analyses, no differences were found in those outcomes when comparing patients with surgical and non-surgical forms of chronic hypoPT or between females and males.

Despite strong evidence of reduced bone remodeling and increased bone mass in patients with chronic hypoPT, the risk of fragility fractures remains unclear. While previous studies suggest no significant overall increase in fracture risk, site-specific differences have been reported. A nationwide health register-based Danish study, including 688 patients with chronic postsurgical hypoPT, found similar risk in patients and matched controls at most fracture sites, including the hip and spine.^13^ However, fractures were 30% less common at the proximal humerus compared to controls. In contrast, the same Danish group reported an increased risk of fractures at the proximal humerus and forearm in 180 patients with non-surgical hypoPT compared to controls.^12^ Conversely, a study from Scotland that included both postsurgical and non-surgical hypoPT found no significant difference in fracture risk compared to matched controls.^11^

Although our study found no increased risk of MOF, we observed a higher risk of vertebral fractures in patients with chronic hypoPT. The reason for this remains unclear, but 1 possible explanation could be detection bias. Regular clinical assessments for hypoPT patients may provide physicians with more opportunities to inquire about and diagnose vertebral fractures, leading to a higher detection rate compared to controls. Additionally, vertebral fractures in controls may be more frequently diagnosed in the primary care setting, which are not captured by the patient register, as it includes only hospital-based diagnoses. However, like our findings, other studies have also reported an increased risk of vertebral fracture in patients with chronic hypoPT, including several smaller studies.^18–20^ A study from Brazil found a higher prevalence of morphometric vertebral fractures in 16 postmenopausal women with postsurgical hypoPT compared to matched controls.^18^ Interestingly, in this small study, vertebral fractures were not limited to individuals with a low bone mass. Notably, 3 of the 4 patients with a BMD T-score above +2.0 had subclinical vertebral fractures, suggesting that BMD may not be a reliable predictor of vertebral fracture risk in chronic hypoPT. Similarly, a recent study from India reported a significantly higher prevalence of vertebral fractures in patients compared to the matched controls, including a greater prevalence of multiple vertebral fractures.^21^ Moreover, a recent systematic review and meta-analysis found a twofold increased risk of vertebral fractures in patients with non-surgical hypoPT.^22^

To explore the increased risk of vertebral fractures in patients with chronic hypoPT despite their higher BMD compared to age and sex matched controls, different measures of bone quality have been investigated. Studies using trabecular bone score (TBS) in this population have shown inconsistent association with fracture risk, leaving the role of TBS in vertebral fractures unresolved.^21^^,^^23^^,^^24^ Other approaches, such as using HR-pQCT to assess bone microarchitecture, or microindentation to measure cortical material bone strength, and hip structural analysis, have provided evidence of altered bone microstructure and strength in hypoPT. However, these findings have not fully explained the mechanisms underlying the increased fracture risk associated with chronic hypoPT.^10^^,^^21^^,^^25^^,^^26^

Patients with vertebral fractures were more likely to have an epilepsy diagnosis than those without vertebral fractures, likely because of the increased risk of falls leading to fractures. However, the absolute number of individuals with epilepsy was small, making it difficult to draw any firm conclusion about the relevance of epilepsy for the increased risk of vertebral fractures.

The reason for the significantly reduced risk of femur fractures in patients with chronic hypoPT in our study is unclear. One potential explanation is the generally higher BMD observed in hypoPT patients, which is a key factor in reducing the risk of non-vertebral fractures. Another possible factor is the significantly higher prevalence of pre-baseline hormone replacement therapy in women with chronic hypoPT compared to control women, which may contribute to improved bone health. Additionally, the standard treatment for hypoPT-calcium supplements and vitamin D, which makes the hypoPT patients less likely to have insufficient intake compared to general population.

The structural and compositional differences between the spine (trabecular-rich) and the hip (cortical-rich) bones may lead to distinct fracture risk profiles in patients with chronic hypoPT. The effect strength of PTH might differ between cortical and trabecular bone. PTH increases bone turnover, however, the effect is more pronounced in cortical bone, leading to cortical thinning and increased porosity. Lack of PTH will therefore have the opposite effect: increased cortical thickness and reduced porosity. In the general population, increased cortical bone is associated with a reduced fracture risk.^27^

Patients with chronic hypoPT were more often diagnosed with osteoporosis than controls. One possible explanation is the higher likelihood of bone mineral measurements in patients with chronic hypoPT, as they have regular contact with healthcare providers. Another possible explanation could be that osteoporosis in the control group is more often diagnosed in the primary care settings by general practitioners, and such diagnoses are therefore not captured in the patient register, which includes only hospital-based data. Additionally, osteoporosis is often underestimated and underdiagnosed in men, which may explain why no significant difference in the osteoporosis diagnosis was observed between males with chronic hypoPT and controls.

Several potential reasons may explain the less frequent dispensation of osteoporosis drugs in patients with chronic hypoPT. One possibility could be the higher pre-baseline use of hormone replacement therapy among women with chronic hypoPT compared to control women. Additionally, the known effect of PTH deficiency in reducing bone remodeling may raise concerns about the use of antiresorptive medications, which further suppress bone turnover. This could lead to uncertainty regarding the efficacy and safety of such treatment in patients with chronic hypoPT.^28^ While epidemiological studies with small sample sizes have sparsely investigated this issue, no studies to date have indicated an increased risk of atypical femoral fractures in patients with chronic hypoPT. Nevertheless, this potential concern may make physicians more cautious in prescribing osteoporosis medications to patients with chronic hypoPT. In conditions marked by low bone formation, such as glucocorticoid-induced osteoporosis, anti-remodeling therapies have demonstrated fracture reduction benefits.^29^

The direct impact of treatment with active vitamin D on risk of fractures remains unclear. While some smaller or short-term studies suggest that calcitriol may help preserve bone density, large-scale evidence showing a significant reduction in fracture risk is limited. A Cochrane review on vitamin D and its analogs in older adults found insufficient proof of a consistent fracture-preventing benefit.^30^

Recently, a new treatment option with a long-acting PTH (palopegteriparatide) has been approved for chronic hypoPT in both Europe and the United States. In a study involving 82 adults with chronic hypoPT, treatment with palopegteriparatide resulted in a decrease in mean BMD Z-scores toward age- and sex-matched norms from baseline to week 52.^31^ Currently, there is no available data on fracture risk in patients treated with palopegterioaratide, but this will be an important area of investigations in the future.

The strengths of this study include the use of a large cohort from high-quality nationwide registries and data linkage to combine ICD codes with prescription drug records to identify patients with chronic hypoPT. Additionally, the concordance between ICD-10 codes and the clinical diagnosis of hypoPT has been validated previously, demonstrating a positive predictive value of 91%.^32^ The proportion of non-surgical hypoPT in this cohort (28%) is similar to what has previously been reported.^33^^,^^34^ There are several limitations. Although patients and controls were matched according to the recorded date of diagnosis, some “early patients,” especially those with non-surgical causes, may have had hypoPT for several years before it was officially documented. Excluding patients with chronic kidney failure (CKD) before start of follow up to avoid including patients with secondary hyperparathyroidism that were incorrectly coded for hypoPT we might have excluded patients with preexisting hypoPT and CKD and therefore underestimate the true fracture risk in the hypoPT population. In the absence of a definitive gold standard for the diagnosis of hypoPT to formally assess accuracy, precision, or reliability of our approach, we propose that the 91% accuracy observed in a Swedish real-world sample from comprehensive national registries likely minimizes the impact of any such bias or imprecision.^32^ We lacked access to bone mineral density measurements, X-rays, or biochemical data from the cases and controls. Since vertebral fractures are sometimes asymptomatic, epidemiological studies based on ICD codes may underestimate the true prevalence, particularly in controls, who are less likely to have frequent interaction with hospital-based health care providers. Further, the diagnostic criteria for vertebral fractures in the clinical settings may vary. No information about physical activity, height, body weight, or smoking was available, but COPD was used as a proxy for heavy smoking. We did not have information regarding bone quality, muscle function, and propensity to falls, which are also of importance to risk of fractures. The study lacked the information on hospital-administered drugs such as zoledronic acid infusions, as SPDR does not capture medication used during inpatient care. Lastly, information on the doses of conventional treatments, including active vitamin D, calcium, or L-thyroxine, was not available in the registries used in this study.

## Conclusion

Patients with chronic hypoPT in Sweden did not exhibit increased risk of MOF. However, they had higher risk of vertebral fractures and a lower risk of femur fractures compared to matched controls. Increased awareness of osteoporosis and vertebral fracture risk in this population is warranted. DXA and vertebral imaging should be considered for patients with chronic hypoPT, especially in those with additional clinical risk factors for osteoporosis, such as thyroid disease, loss of height, female gender, or older age, as vertebral fractures can be asymptomatic and do not always come to clinical attention.

## Supplementary Material

Supplementary_Table_1_MS_ASBMR-24121065_R_zjaf0611

Supplementary_Table_2_MS_ASBMR-24121065_R1_zjaf061

Supplementary_Table_3_MS_ASBMR-24121065_R1_zjaf061

Supplementary_Table_4_MS_ASBMR-24121065_R1_zjaf061

## Data Availability

Data available on request.

## References

[1] Clarke BL, Brown EM, Collins MT, et al. Epidemiology and diagnosis of hypoparathyroidism. J Clin Endocrinol Metab. 2016;101(6):2284–2299. 26943720 10.1210/jc.2015-3908 PMC5393595

[2] Harslof T, Rolighed L, Rejnmark L. Huge variations in definition and reported incidence of postsurgical hypoparathyroidism: a systematic review. Endocrine. 2019;64(1):176–183. 30788669 10.1007/s12020-019-01858-4

[3] Bergenfelz A, Jansson S, Kristoffersson A, et al. Complications to thyroid surgery: results as reported in a database from a multicenter audit comprising 3,660 patients. Langenbeck's Arch Surg. 2008;393(5):667–673. 18633639 10.1007/s00423-008-0366-7

[4] Chadwick DR . Hypocalcaemia and permanent hypoparathyroidism after total/bilateral thyroidectomy in the BAETS registry. Gland Surg. 2017;6(Suppl 1):S69–S74. 29322024 10.21037/gs.2017.09.14 PMC5756750

[5] Khan AA, Bilezikian JP, Brandi ML, et al. Evaluation and Management of Hypoparathyroidism Summary Statement and Guidelines from the second international workshop. J Bone Miner Res. 2022;37(12):2568–2585. 36054621 10.1002/jbmr.4691

[6] Silva BC, Bilezikian JP. Skeletal abnormalities in hypoparathyroidism and in primary hyperparathyroidism. Rev Endocr Metab Disord. 2021;22(4):789–802. 33200346 10.1007/s11154-020-09614-0

[7] Rubin MR, Dempster DW, Sliney J Jr, et al. PTH(1-84) administration reverses abnormal bone-remodeling dynamics and structure in hypoparathyroidism. J Bone Miner Res. 2011;26(11):2727–2736. 21735476 10.1002/jbmr.452 PMC4019384

[8] Langdahl BL, Mortensen L, Vesterby A, Eriksen EF, Charles P. Bone histomorphometry in hypoparathyroid patients treated with vitamin D. Bone. 1996;18(2):103–108. 8833203 10.1016/8756-3282(95)00443-2

[9] Agarwal S, McMahon DJ, Chen J, et al. The clinical and skeletal effects of long-term therapy of hypoparathyroidism with rhPTH(1-84). J Bone Miner Res. 2023;38(4):480–492. 36726204 10.1002/jbmr.4780 PMC10101915

[10] Starr JR, Tabacco G, Majeed R, Omeragic B, Bandeira L, Rubin MR. PTH and bone material strength in hypoparathyroidism as measured by impact microindentation. Osteoporos Int. 2020;31(2):327–333. 31720712 10.1007/s00198-019-05177-2

[11] Vadiveloo T, Donnan PT, Leese CJ, Abraham KJ, Leese GP. Increased mortality and morbidity in patients with chronic hypoparathyroidism: a population-based study. Clin Endocrinol. 2019;90(2):285–292. 10.1111/cen.13895 30375660

[12] Underbjerg L, Sikjaer T, Mosekilde L, Rejnmark L. The epidemiology of nonsurgical hypoparathyroidism in Denmark: a nationwide case finding study. J Bone Miner Res. 2015;30(9):1738–1744. 25753591 10.1002/jbmr.2501

[13] Underbjerg L, Sikjaer T, Mosekilde L, Rejnmark L. Postsurgical hypoparathyroidism--risk of fractures, psychiatric diseases, cancer, cataract, and infections. J Bone Miner Res. 2014;29(11):2504–2510. 24806578 10.1002/jbmr.2273

[14] Laugesen K, Ludvigsson JF, Schmidt M, et al. Nordic health registry-based research: a review of health care systems and key registries. Clin Epidemiol. 2021;13:533–554. 34321928 10.2147/CLEP.S314959 PMC8302231

[15] Wettermark B, Hammar N, Fored CM, et al. The new Swedish prescribed drug register--opportunities for pharmacoepidemiological research and experience from the first six months. Pharmacoepidemiol Drug Saf. 2007;16(7):726–735. 16897791 10.1002/pds.1294

[16] Ludvigsson JF, Almqvist C, Bonamy AK, et al. Registers of the Swedish total population and their use in medical research. Eur J Epidemiol. 2016;31(2):125–136. 26769609 10.1007/s10654-016-0117-y

[17] Kanis JA, McCloskey EV, Johansson H, Oden A, Strom O, Borgstrom F. Development and use of FRAX in osteoporosis. Osteoporos Int. 2010;21(Suppl 2):S407–S413. 20464374 10.1007/s00198-010-1253-y

[18] Mendonca ML, Pereira FA, Nogueira-Barbosa MH, et al. Increased vertebral morphometric fracture in patients with postsurgical hypoparathyroidism despite normal bone mineral density. BMC Endocr Disord. 2013;13(1):1. 10.1186/1472-6823-13-1 PMC3546901 23286605

[19] Kim SH, Rhee Y, Kim YM, et al. Prevalence and complications of nonsurgical hypoparathyroidism in Korea: a nationwide cohort study. PLoS One. 2020;15(5):e0232842. 32384131 10.1371/journal.pone.0232842 PMC7209335

[20] Chawla H, Saha S, Kandasamy D, Sharma R, Sreenivas V, Goswami R. Vertebral fractures and bone mineral density in patients with idiopathic hypoparathyroidism on long-term follow-up. J Clin Endocrinol Metab. 2017;102(1):251–258. 27813708 10.1210/jc.2016-3292

[21] Saha S, Mannar V, Kandasamy D, Sreenivas V, Goswami R. Vertebral fractures, trabecular bone score and their determinants in chronic hypoparathyroidism. J Endocrinol Investig. 2022;45(9):1777–1786. 35585296 10.1007/s40618-022-01818-2

[22] Pal R, Bhadada SK, Mukherjee S, Banerjee M, Kumar A. Fracture risk in hypoparathyroidism: a systematic review and meta-analysis. Osteoporos Int. 2021;32(11):2145–2153. 34021765 10.1007/s00198-021-05966-8

[23] Cipriani C, Minisola S, Bilezikian JP, et al. Vertebral fracture assessment in postmenopausal women with postsurgical hypoparathyroidism. J Clin Endocrinol Metab. 2021;106(5):1303–1311. 33567075 10.1210/clinem/dgab076 PMC8063231

[24] Sakane EN, Vieira MCC, Lazaretti-Castro M, Maeda SS. Predictors of poor bone microarchitecture assessed by trabecular bone score in postsurgical hypoparathyroidism. J Clin Endocrinol Metab. 2019;104(12):5795–5803. 31305931 10.1210/jc.2019-00698

[25] Cusano NE, Nishiyama KK, Zhang C, et al. Noninvasive assessment of skeletal microstructure and estimated bone strength in hypoparathyroidism. J Bone Miner Res. 2016;31(2):308–316. 26234545 10.1002/jbmr.2609 PMC4832602

[26] Chen Q, Kaji H, Iu MF, et al. Effects of an excess and a deficiency of endogenous parathyroid hormone on volumetric bone mineral density and bone geometry determined by peripheral quantitative computed tomography in female subjects. J Clin Endocrinol Metab. 2003;88(10):4655–4658. 14557436 10.1210/jc.2003-030470

[27] Duan Y, De Luca V, Seeman E. Parathyroid hormone deficiency and excess: similar effects on trabecular bone but differing effects on cortical bone. J Clin Endocrinol Metab. 1999;84(2):718–722. 10022443 10.1210/jcem.84.2.5498

[28] Meier RP, Perneger TV, Stern R, Rizzoli R, Peter RE. Increasing occurrence of atypical femoral fractures associated with bisphosphonate use. Arch Intern Med. 2012;172(12):930–936. 22732749 10.1001/archinternmed.2012.1796

[29] Saag KG, Wagman RB, Geusens P, et al. Denosumab versus risedronate in glucocorticoid-induced osteoporosis: a multicentre, randomised, double-blind, active-controlled, double-dummy, non-inferiority study. Lancet Diabetes Endocrinol. 2018;6(6):445–454. 29631782 10.1016/S2213-8587(18)30075-5

[30] Avenell A, Mak JC, O'Connell D. Vitamin D and vitamin D analogues for preventing fractures in post-menopausal women and older men. Cochrane Database Syst Rev. 2014, 2014;(4):CD000227. 24729336 10.1002/14651858.CD000227.pub4 PMC7032685

[31] Clarke BL, Khan AA, Rubin MR, et al. Efficacy and safety of TransCon PTH in adults with hypoparathyroidism: 52-week results from the phase 3 PaTHway trial. J Clin Endocrinol Metab. 2025;110(4):951-960. 10.1210/clinem/dgae693 PMC11913112 39376010

[32] Kamal W, Bjornsdottir S, Kampe O, Trolle LY. Concordance between ICD-10 codes and clinical diagnosis of hypoparathyroidism in Sweden. Clin Epidemiol. 2020;12:327–331. 32273771 10.2147/CLEP.S242528 PMC7102876

[33] Astor MC, Lovas K, Debowska A, et al. Epidemiology and health-related quality of life in hypoparathyroidism in Norway. J Clin Endocrinol Metab. 2016;101(8):3045–3053. 27186861 10.1210/jc.2016-1477 PMC4971340

[34] Powers J, Joy K, Ruscio A, Lagast H. Prevalence and incidence of hypoparathyroidism in the United States using a large claims database. J Bone Miner Res. 2013;28(12):2570–2576. 23737456 10.1002/jbmr.2004

