# Supplementary material for: Increased risk of vertebral fractures and reduced risk of femur fractures in patients with chronic hypoparathyroidism: a nationwide cohort study in Sweden
Source: J Bone Miner Res. 2025 May 5;40(7):860–7. doi: 10.1093/jbmr/zjaf061 (PMC12188750; doi:10.1093/jbmr/zjaf061)
Supplement: Supplementary_Table_1_MS_ASBMR-24121065_R_zjaf0611 [file supplementary_table_1_ms_asbmr-24121065_r_zjaf0611.docx]

Supplementary Table 1. ICD-10 diagnoses for kidney failure excluded at baseline or within one year from baseline.

|  | ICD-10 diagnoses at baseline |
| --- | --- |
| Type 1 diabetes mellitus with kidney complications | E10.2 |
| Type 2 diabetes mellitus with kidney complications | E11.2 |
| Hypertensive chronic kidney disease | I12.0 and I12.9 |
| Glomerular diseases | N00-08 |
| Renal tubulo-interstitial diseases | N10-16, except Acute tubulo-interstitial nephritis N10.9 |
| Chronic kidney disease and unspecified kidney failure | N18-19 |
| Other disorders of kidney and ureter | N25-29 |
| Cystic kidney disease | Q61 |
| Encounter for care involving renal dialysis | Z49 |
| Dependence on renal dialysis | Z99.2 |
| Kidney transplant status | Z94.0 |
