# Supplementary material for: Increased risk of vertebral fractures and reduced risk of femur fractures in patients with chronic hypoparathyroidism: a nationwide cohort study in Sweden
Source: J Bone Miner Res. 2025 May 5;40(7):860–7. doi: 10.1093/jbmr/zjaf061 (PMC12188750; doi:10.1093/jbmr/zjaf061)
Supplement: Supplementary_Table_2_MS_ASBMR-24121065_R1_zjaf061 [file supplementary_table_2_ms_asbmr-24121065_r1_zjaf061.docx]

Supplementary Table 2. ICD-10 codes of comorbidities related to increased fracture risk.

| Comorbidities | ICD-10 | ATC |
| --- | --- | --- |
| Type 1 diabetes | E10* |  |
| Type 2 diabetes | E11* |  |
| Malignant neoplasms | C00-96* |  |
| Thyrotoxicosis | E05* |  |
| Ischemic heart diseases | I20-I25* |  |
| Cerebrovascular diseases | I60-I69* |  |
| Chronic kidney disease | N18* |  |
| Diseases of liver | K70-K77* |  |
| Rheumatoid arthritis | M05-M06* |  |
| Chronic obstructive pulmonary disease (COPD) | J44* | N07BA** |
| Alcohol related disorders | F10* | N07BB*** |

* All subcodes. ** Drugs used in nicotine dependence. *** Drugs used in

alcohol dependence.
