# Supplementary material for: Increased risk of vertebral fractures and reduced risk of femur fractures in patients with chronic hypoparathyroidism: a nationwide cohort study in Sweden
Source: J Bone Miner Res. 2025 May 5;40(7):860–7. doi: 10.1093/jbmr/zjaf061 (PMC12188750; doi:10.1093/jbmr/zjaf061)
Supplement: Supplementary_Table_3_MS_ASBMR-24121065_R1_zjaf061 [file supplementary_table_3_ms_asbmr-24121065_r1_zjaf061.docx]

Supplementary Table 3 ICD-10-codes for fractures

| Any MOF | S12, Fracture of cervical vertebra and other parts of neck  S22.0, Fracture of thoracic vertebra  S22.1, Multiple fractures of thoracic spine  S32.0 Fracture of lumbar vertebra  S32.7, Multiple fractures of lumbar spine and pelvis  S42.2, Fracture of upper end of humerus  S42.3, Fracture of shaft of humerus  S42.4, Fracture of lower end of humerus  S42.7, Multiple fractures of clavicle, scapula and humerus  S52.5, Fracture of lower end of radius  S52.6, Fracture of lower end of ulna  S72.0, Fracture of head and neck of femur  S72.1, Pertrochanteric fracture  S72.2, Subtrochanteric fracture of femur  T02.1, Fractures involving thorax with lower back and pelvis  T08, Fracture of spine, level unspecified |
| --- | --- |
| Fracture of spine | S12, S22.0, S22.1, S32.0, S32.7, T02.1, T08 |
| Fracture of hip | S72.0, S72.1, S72.2 |
| Fracture of humerus | S42.2, S42.3, S42.4, S42.7 |
| Fracture of lower end of radius | S52.5, S52.6 |
