# Supplementary material for: Increased risk of vertebral fractures and reduced risk of femur fractures in patients with chronic hypoparathyroidism: a nationwide cohort study in Sweden
Source: J Bone Miner Res. 2025 May 5;40(7):860–7. doi: 10.1093/jbmr/zjaf061 (PMC12188750; doi:10.1093/jbmr/zjaf061)
Supplement: Supplementary_Table_4_MS_ASBMR-24121065_R1_zjaf061 [file supplementary_table_4_ms_asbmr-24121065_r1_zjaf061.docx]

Supplementary Table 4. ATC-codes prescriptions for osteoporosis treatment.

| ATC-code | Active substance |
| --- | --- |
| M05BA01 | Etidronate |
| M05BB01 | Etidronate and calcium |
| M05BA02 | Clodronic acid |
| M05BA03 | Pamidronic acid |
| M05BA04 | Alendronic acid |
| M05BB03 | Alendronic acid and cholecalciferol |
| M05BB05 | Alendronic acid, calcium and cholecalciferol |
| M05BB06 | Alendronic acid and alfakalcidol |
| M05BA05 | Tiludronic acid |
| M05BA06 | Ibandronic acid |
| M05BA07 | Risedronic acid |
| M05BB02 | Risedronic acid and calcium |
| M05BB04 | Risedronic acid, calcium and cholecalciferol |
| M05BB07 | Risedronic acid and cholecalciferol |
| M05BA08 | Zoledronic acid |
| M05BB08 | Zoledronic acid, calcium and cholecalciferol |
| M05BX03 | Strontium ranelate |
| M05BX53 | Strontium ranelate and cholecalciferol |
| M05BX04 | Denosumab |
| M05BX06 | Romosozumab |
| H05AA02 | Teriparatide |
| H05AA04 | Abaloparatide |
| G03XC01 | Raloxifene |
